# The Mechanisms of Imatinib Resistance in Gastrointestinal Stromal Tumours: Theoretical Basis and Therapeutic Aspect

**DOI:** 10.1111/jcmm.70931

**Published:** 2025-11-13

**Authors:** Nuerbiye Abudurexiti, Yidan Lou, Mengqi Wu, Yingchen Huang, Shan Huang, Kaibo Guo, Song Zheng

**Affiliations:** ^1^ The Fourth School of Clinical Medicine Zhejiang Chinese Medical University Hangzhou China; ^2^ Department of Oncology, Affiliated Hangzhou First People's Hospital Westlake University Hangzhou China; ^3^ Key Laboratory of Clinical Cancer Pharmacology & Toxicology Research of Zhejiang Province, Affiliated Hangzhou First People's Hospital Westlake University Hangzhou China; ^4^ Zhejiang University School of Medicine Hangzhou China; ^5^ Hangzhou Traditional Chinese Medicine Hospital of Zhejiang Chinese Medical University Hangzhou China

**Keywords:** drug resistance, gastrointestinal stromal tumours, imatinib, target therapy, tyrosine kinase inhibitor

## Abstract

Imatinib is a small molecule that inhibits receptor tyrosine kinases KIT and platelet‐derived growth factor receptor α (PDGFRA). It was approved by the U.S. Food and Drug Administration (FDA) in 2002 for the treatment of advanced, unresectable gastrointestinal stromal tumours (GISTs). Since then, additional kinase inhibitors targeting multiple pathways have also been introduced. However, as treatment with imatinib is prolonged, its effectiveness gradually declines, with 50% of patients experiencing relapse within 2–5 years. This decline in effectiveness is largely attributed to the development of both initial and acquired resistance. Due to these mechanisms of resistance, many advanced GIST patients struggle to achieve long‐term benefits from systemic treatment. This review aims to summarise the mechanisms of resistance to imatinib and their relationship with GIST molecular subtypes, while also exploring the latest strategies to overcome resistance. We discuss the alterations in signalling pathways following imatinib resistance, the disruption of autophagy and glycolysis, the role of microRNAs and immunotherapy in resistant GISTs, and combination therapies. Furthermore, we examine ongoing clinical trials and potential future therapies that could be integrated into clinical practice, offering guidance for new treatment strategies that aim to enhance the prognosis of advanced GIST patients.

**Abbreviations**
18F‐FDG‐PET: 18F‐fluorodeoxyglucose positron emission tomography
4E‐BP1: eukaryotic translation initiation factor 4E‐binding protein 1
ABL: Abelson murine leukaemia viral oncogene homologue 1
ACK1: activated Cdc42‐associated kinase 1
ARG: Abl‐related gene
ATG5: autophagy‐related protein 5
ATP: adenosine triphosphate
BCR‐ABL: breakpoint cluster region–Abelson fusion gene
BECN1: Beclin‐1
BRAF: B‐Raf proto‐oncogene, serine/threonine kinase
CCDC26: coiled‐coil domain‐containing 26
CD8+: cluster of differentiation 8 positive T lymphocytes
circRNAs: circular RNAs
CTLA‐4: Cytotoxic T‐lymphocyte–associated protein 4
DEPDC5: DEP domain‐containing protein 5
ERK: extracellular signal‐regulated kinase
FGF: fibroblast growth factor
GDNF: glial cell line‐derived neurotrophic factor
GFRA1: GDNF family receptor alpha‐1
GISTs: gastrointestinal stromal tumours
GLUT1: glucose transporter 1
JM: juxtamembrane domain
KIT: KIT proto‐oncogene receptor tyrosine kinase (CD117)
KRAS: Kirsten rat sarcoma viral oncogene homologue
LC3‐II/I: microtubule‐associated protein 1 light chain 3, isoforms II/I
MAPK: mitogen‐activated protein kinase
MCOLN1: mucolipin‐1 (TRPML1 channel)
mTOR: mechanistic target of rapamycin
mTORC1: mechanistic target of rapamycin complex 1
p70S6K: ribosomal protein S6 kinase 1(S6K1)
PARP: poly(ADP‐ribose) polymerase
PD‐1: programmed cell death protein 1
PDCD4: programmed cell death 4
PD‐L1: programmed cell death ligand 1
PI3K: phosphatidylinositol 3‐kinase
PTEN: phosphatase and tensin homologue
PTPN18: protein tyrosine phosphatase, non‐receptor type 18
RAF: rapidly accelerated fibrosarcoma kinase family
RTK: receptor tyrosine kinase
RT‐PCR: reverse transcription polymerase chain reaction
S6: ribosomal protein S6
S6K: ribosomal protein S6 kinase beta‐1
SCF: stem cell factor
SDH: succinate dehydrogenase
SQSTM1: equestosome 1 (p62)
TFEB: transcription factor EB
TK: tyrosine kinase
VEGFR: vascular endothelial growth factor receptor
VLX600: iron‐chelating mitochondrial oxidative

## 1 Introduction

Gastrointestinal stromal tumours (GISTs) are the most common mesenchymal tumours of the gastrointestinal tract, with approximately 20%–30% of GIST patients demonstrating malignant behaviour [1]. GISTs primarily originate from the precursors of Cajal interstitial cells (ICCs), which act as pacemakers for peristaltic contractions in the gastrointestinal tract. The global annual incidence of GIST is 10 per million, with the highest rates observed in Shanghai, Hong Kong, and Taiwan, reaching 19–22 per million annually, and approximately 15,000 new GIST cases are diagnosed worldwide each year [2, 3]. GIST can occur at any age, with the median onset age around 60 years, and the incidence is similar in both men and women. Histologically, GISTs are categorised into three primary types: spindle cell, epithelioid, and mixed. The majority of these tumours are found in the stomach (60%–70%), followed by the small intestine (20%–25%), while those in the colon, oesophagus, and extra‐gastrointestinal sites are less frequent [4, 5].

The pathogenesis of GISTs is driven by gain‐of‐function mutations in receptor tyrosine kinase KIT (70%) and platelet‐derived growth factor receptor α (PDGFRA) (15%) [6, 7]. In 15% of GIST cases, no mutations in KIT or PDGFRA are found, referred to as ‘KP‐wild‐type GISTs’ which represent 85% of paediatric GISTs. The primary subtype of wild‐type GIST is SDH‐deficient GIST, along with mutations in genes such as NF1, KRAS and BRAF [8, 9, 10].

Prior to the approval of imatinib for GIST treatment, GISTs were known to be resistant to conventional chemotherapy, and the treatment of primary localised GISTs relied mainly on surgical resection. Targeted therapy is the most crucial treatment strategy for advanced GIST. Imatinib is a Type II tyrosine kinase inhibitor (TKI) that targets the inactive conformation of KIT, PDGFR, BCR‐ABL, ABL, and ARG. Over the past two decades, TKI therapy has increased the median survival time of advanced GIST patients from 18 months to more than 5 years, revolutionising the prognosis of GIST patients [11, 12]. After treatment with imatinib, 15%–20% of patients attain stable disease, 65%–70% have partial remission, but regrettably, only 5% or fewer achieve complete remission. Secondary resistance occurs in 40%–50% of cases within 2–5 years of treatment [13]. Sunitinib [14], a multi‐target Type I TKI inhibiting KIT, PDGFRs, VEGFRs, and RET, was approved in 2006 and is used as a second‐line therapy for imatinib‐resistant GIST. Regorafenib [15], a broader‐spectrum Type II TKI targeting KIT, RET, RAF, and angiogenic receptors (VEGFR1‐3, TIE2), was approved in 2012 and is indicated as a third‐line therapy for patients following sunitinib progression. However, the median progression‐free survival (PFS) following sunitinib treatment is 6.3 months, whereas regorafenib provides only 4.8 months [14, 16]. Ripretinib, a novel Type II TKI specifically designed to target a broad spectrum of KIT and PDGFRA mutations by switching the kinase back to its inactive state, approved in 2020, is recommended for GIST patients who have received at least three prior kinase inhibitors, positioning it as a fourth‐line treatment option [17]. Among patients resistant to imatinib, 78% are also resistant to sunitinib, whereas 70% maintain sensitivity to ripretinib [18]. While the efficacy of ripretinib in GIST is similar to that of sunitinib, it demonstrates better tolerance and safety [19, 20, 21].

Imatinib resistance arises from various resistance mechanisms. Primary resistance appears within 3–6 months of initiating imatinib treatment, with disease progression associated with GIST molecular subtypes, especially in PDGFRA D842V mutations and wild‐type GISTs [22]. Acquired resistance represents the primary challenge, contributing up to 70% of cases, primarily driven by post‐treatment genomic alterations in KIT or PDGFRA kinase domains [23]. The dominant mechanism involves mutational changes within the KIT kinase domain, frequently localised to the ATP binding site or activation loop regions [24]. Studies have shown that inhibiting KIT does not eliminate all GIST cells; rather, many cells survive and enter a non‐proliferative dormant state, remaining dormant for years [25]. Moreover, dormant cells can retain vitality, particularly tumour cells with impaired checkpoint control that may re‐enter the cell cycle, leading to disease progression and eventual acquired resistance and treatment failure [26, 27]. This has been confirmed in recent clinical trials, where patients who interrupt imatinib treatment show shorter overall survival and faster development of imatinib resistance compared to those on continuous oral imatinib [28, 29, 30].

**FIGURE 1:**
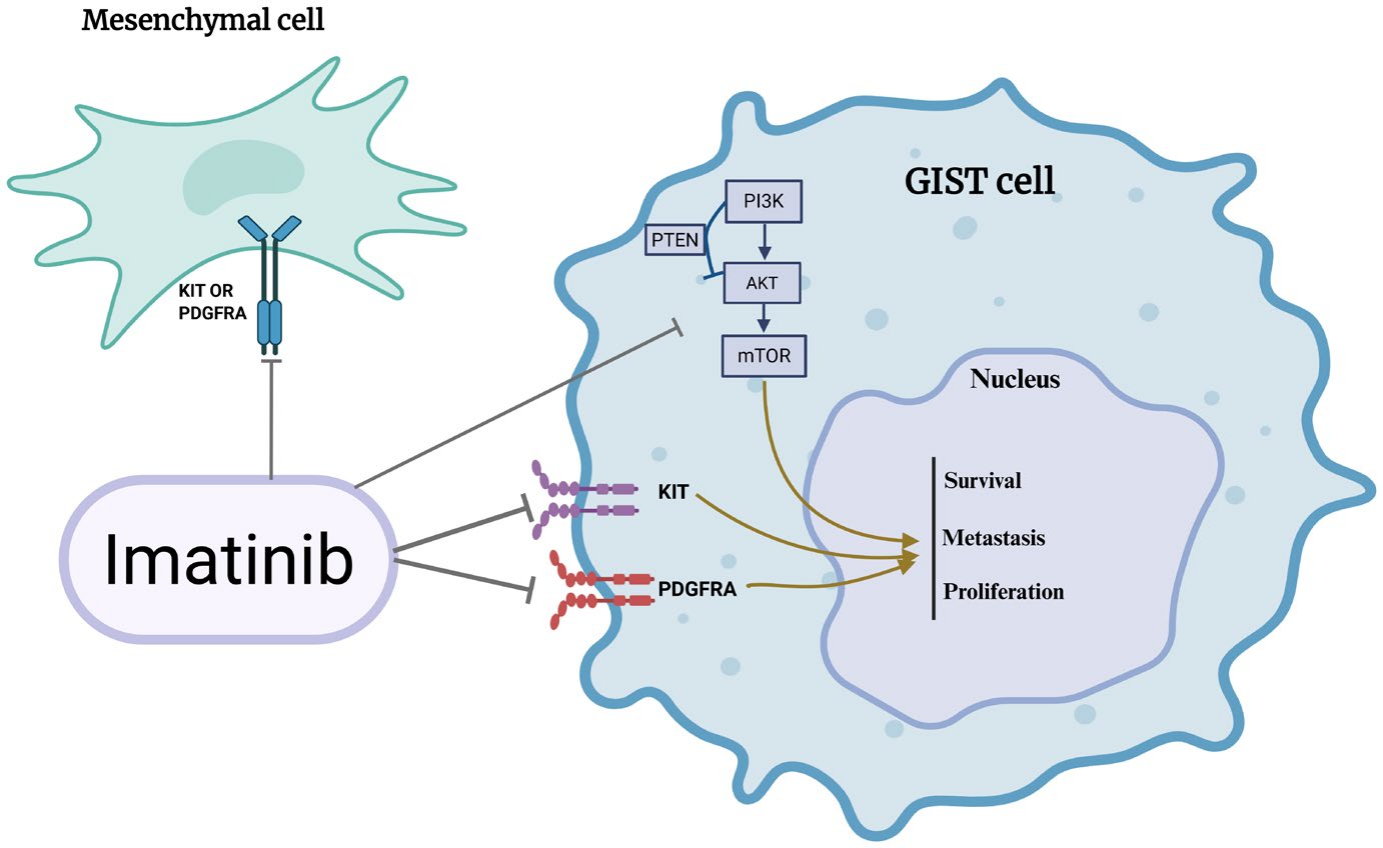
GIST pathogenesis and imatinib mechanism of action. GIST cell originates from a mesenchymal cell and is driven by mutations in the KIT or PDGFRA genes. These activated receptors trigger the PI3K/AKT/mTOR signalling cascade (inhibited by PTEN), promoting cell survival, proliferation, and metastasis. The drug Imatinib works by inhibiting the KIT and PDGFRA receptors to block this oncogenic signalling.

In conclusion, before the advent of imatinib, the prognosis for patients with advanced GIST was dismal. However, the introduction of targeted therapies has significantly improved outcomes for these patients. KIT secondary mutations are now recognised as the primary cause of the failure of imatinib‐targeted therapy. Resistance occurs due to various factors, making overcoming imatinib resistance a daunting challenge. Therefore, in this review, we discuss the major factors involved in imatinib resistance, including molecular subtypes, signalling pathways, metabolic factors, immune evasion, emerging combination therapies to overcome resistance, and their limitations (Figure 1).

## 2 Molecular Subtypes of GIST and Relationship With Imatinib Resistance

The sensitivity of tumours to imatinib depends on the initial mutation type and is closely related to their resistance. Therefore, activating mutations in KIT/PDGFRA form the theoretical basis for TKI therapy and the development of new drugs [31, 32, 33, 34, 35]. GISTs can be broadly categorised into KIT‐mutant, PDGFRA‐mutant, and KIT/PDGFRA wild‐type (WT) based on mutations in driver genes. These mutations result in receptor self‐activation without ligands, activating pathways that inhibit apoptosis and promote cell proliferation and cell cycle activation, facilitating tumorigenesis [35, 36].

The c‐KIT gene encodes a type III receptor tyrosine kinase (RTK) that is activated upon binding with the SCF ligand. This activation results in continuous receptor tyrosine kinase activation, which drives the development of GIST [37, 38]. GISTs with KIT mutations are generally sensitive to imatinib, and even in advanced stages or in cases of resistance, KIT remains a central oncogenic driver [39]. However, current KIT kinase inhibitors are ineffective against all relevant resistance mutations, limiting their use as standalone therapies. KIT mutations, which occur de novo, are found in four exons: exon 11, encoding the JM region (70% of all GISTs), exon 9 encoding the extracellular domain (10%), exon 13 encoding TK domain I (ATP‐binding pocket) (1%), and exon 17 encoding TK domain II (activation loop) (1%) [40, 41, 42]. GISTs with exon 11 mutations are highly responsive to imatinib, but they are also the most common in cases of secondary resistance. A phase II study of advanced GIST found that 67% of tumours from imatinib‐resistant patients had new or secondary KIT mutations, with exon 11 mutations being the most frequent [41, 43, 44]. KIT exon 9 mutations show poor responses to imatinib, resulting in a worse prognosis for these patients [32, 45, 46]. Reports suggest that primary resistance rates to imatinib for KIT exon 11, KIT exon 9, and wild‐type GISTs are 5%, 16%, and 23%, respectively [47]. Mutations in exons 13 and 17 are extremely rare but are commonly seen in secondary mutations [40]. In a phase III clinical trial of imatinib‐resistant patients, sunitinib showed improved PFS for GIST patients with KIT exon 11 + 13/14 mutations, while ripretinib provided greater benefits for those with KIT exon 11 + 17/18 mutations [48, 49].

PDGFRA‐mutant GISTs primarily occur in the stomach (90%–93%), with 80% of mutations found in exon 18. The p.D842V mutation in exon 18 accounts for 60%–65% of all PDGFRA mutations in GISTs. This mutation stabilises the kinase domain in an active conformation, preventing imatinib binding and causing primary resistance. For decades, no effective treatments were available for this subtype, earning it the reputation of being the ‘bad apple’ in GISTs [50]. Interestingly, this subtype responds well to avapritinib therapy. In January 2020, the FDA approved avapritinib for the treatment of adult patients with inoperable or metastatic PDGFRA exon 18‐mutant GIST, including those with the PDGFRA D842V mutation [51, 52]. Other PDGFRA mutation subtypes are sensitive to imatinib [53, 54, 55].

**FIGURE 2:**
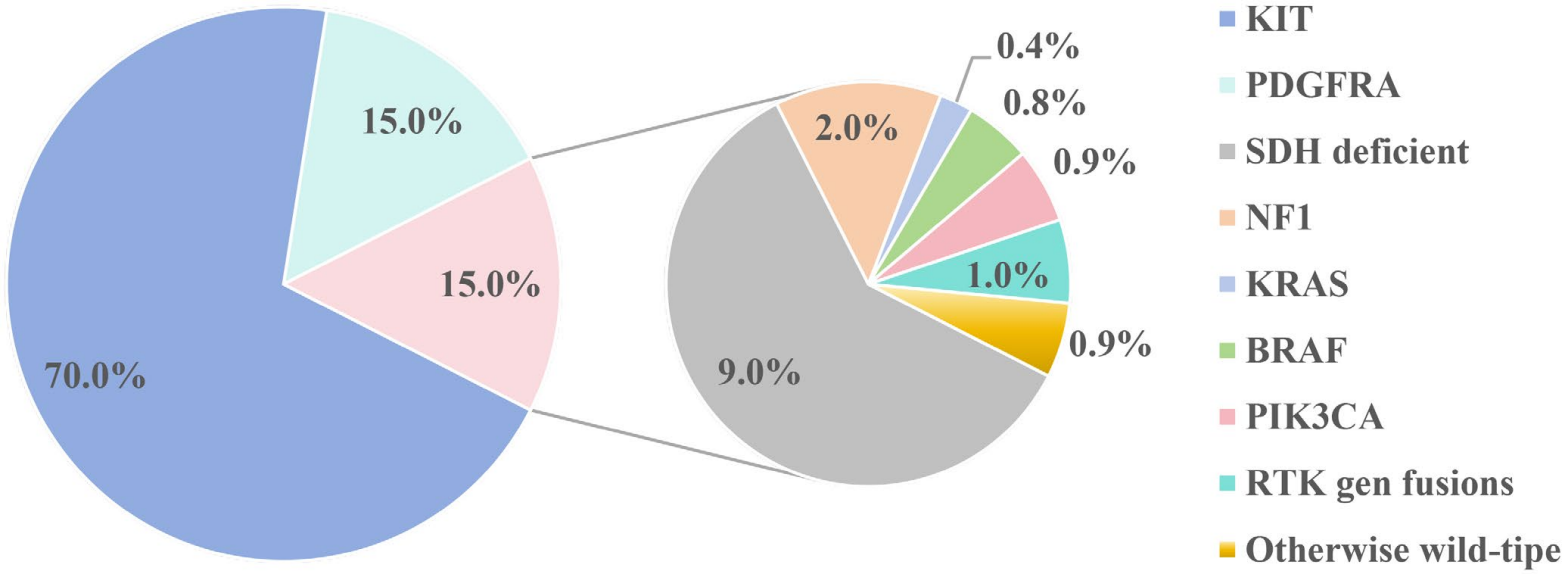
Molecular subtypes and mutation prevalence in GIST. This pie chart illustrates the mutation spectrum in GIST. The vast majority (70%) are driven by KIT mutations, followed by PDGFRA (15%) and SDH‐deficient subtypes (15%). The remaining rare subtypes, including those involving NF1, KRAS, BRAF, PIK3CA, and RTK fusions, collectively account for a very small proportion (around 5%) of cases.

KP‐wtGISTs represents about 15% of all GIST cases and is categorised into two subgroups: SDH‐deficient GIST and non‐SDH‐deficient GIST [56, 57, 58]. SDH‐deficient GIST, which lacks gain‐of‐function mutations in KIT and PDGFRA, is typically considered primary resistant to TKIs [59, 60]. The SDH enzyme is a mitochondrial complex composed of four subunits (SDHA, SDHB, SDHC, and SDHD) that plays a dual role in both the tricarboxylic acid (TCA) cycle and the electron transport chain. Loss of SDH function leads to succinate accumulation, which promotes tumorigenesis through multiple mechanisms. As a result, SDH‐deficient GIST is typically considered primarily resistant to TKIs [59, 60]. But, It responds well to regorafenib, and its clinical outcomes are superior to those of other KP‐wtGISTs [56]. Nevertheless, the treatment outcomes for advanced SDH‐deficient GIST patients are significantly worse than those for KIT‐mutant GIST patients, highlighting the need for more effective treatment strategies. SDH‐deficient GISTs typically exhibit a high tumour mutational burden and abundant tumour‐infiltrating lymphocytes, creating a favourable microenvironment for immunotherapy. Metabolism‐targeting agents may represent potential therapeutic targets. Although the mechanisms underlying SDH deficiency are complex, they give rise to multiple vulnerable points—such as metabolic, epigenetic, and immune dysregulation—that are amenable to intervention. Targeted therapies addressing these vulnerabilities are currently a major focus of research and have shown preliminary promise in clinical settings (Figure 2).

## 3 Molecular Alterations in GIST Pathogenesis and Imatinib Resistance

### 3.1 PI3K/AKT/mTOR Signalling Pathway

The majority of GISTs with acquired secondary KIT mutations feature reactivation of KIT downstream pathways [61]. The PI3K/AKT/mTOR pathway is one of the major downstream components of the KIT gene [62, 63]. In imatinib‐resistant GISTs, the PI3K/AKT/mTOR pathway is highly active [64, 65, 66]. The activation rate of AKT/mTOR in secondary imatinib‐resistant GISTs (53.1%) is significantly higher than in imatinib‐sensitive GISTs (27.1%) and primary resistant GISTs (33.3%) [67], which is a result of secondary mutations in the KIT or PDGFRA kinase domains [68]. Long‐term exposure of imatinib‐resistant cell lines leads to epigenetic silencing of PTEN, causing excessive activation of the PI3K/AKT pathway [69, 70]. ACK1 serves as a critical upstream activator of the PI3K/AKT signalling pathway, capable of alternatively sustaining constitutive activation of this pathway following KIT inhibition. Upon the development of acquired resistance to imatinib in tumours, the expression and/or activity of ACK1 is significantly upregulated. In both imatinib‐sensitive and imatinib‐resistant GIST cell lines, treatment with the specific ACK1 inhibitor AIM‐100 or ACK1 siRNA significantly inhibits cell migration, which correlates with the inactivation of the PI3K/AKT/mTOR and RAF/MAPK signalling pathways [24]. Furthermore, in vivo experiments show that blocking PD‐1/PD‐L1 through the PI3K/AKT/mTOR pathway in PD‐L1‐high expressing GISTs rescues exhausted CD8+ T cells, enhancing imatinib efficacy [71]. Consequently, drugs targeting this pathway, including PI3K inhibitors, Akt inhibitors, dual PI3K/mTOR inhibitors, and combinations with imatinib, are extensively studied to reverse imatinib resistance in GISTs.

PI3K is crucial for ICC cell proliferation and GIST tumorigenesis, as the ligand‐independent activation of KIT due to mutations depends on PI3K and KIT interaction. Disruption of PI3K binding to downstream molecules inhibits KIT's ligand‐independent activation, thereby increasing its sensitivity to imatinib [42, 44, 72, 73, 74]. In double‐mutant KitV558Δ; Y567F/Y567F knock‐in mice, where SRC family kinase binding sites (pY567) are absent, the MAPK signalling pathway is weakened while the PI3K pathway is activated, promoting GIST resistance [72]. In in vitro and in vivo experiments, the effects of PI3K inhibitors as monotherapy and in combination with imatinib were evaluated. PI3K inhibitors alone showed antitumor effects, and combination therapy significantly reduced tumour volume through apoptosis induction [75, 76, 77]. However, in a phase Ib multicenter study involving 60 patients, buparlisib (pan‐PI3K Type I inhibitor) combined with treatment for advanced GIST resulted in 58.3% of patients experiencing grade 3 or higher adverse events (AEs), and the combination treatment showed no significant synergistic effect [78]. In a multicenter phase Ib clinical study involving 56 GIST patients resistant to imatinib and sunitinib, the PI3K inhibitor alpelisib combined with imatinib also failed due to lack of additional benefits and excessive adverse reactions [79]. The most common AEs were gastrointestinal disorders, hyperglycemia, skin rash, and mood disturbances.

AKT activation is a known marker of imatinib resistance in GISTs [64, 67]. AKT inhibitors specifically block AKT activation, inhibit mTORC1 activation, and regulate the downstream effects of the PI3K signalling cascade. Studies have shown that combining imatinib with the allosteric AKT inhibitor MK‐2206 (Type II) results in significant synergy in both imatinib‐sensitive and resistant GIST cells, as well as in xenograft animal models [80]. In both in vitro and in vivo studies, the combination of the ATP‐competitive AKT inhibitor MK‐4440 (Type I) and imatinib led to an increase in PDCD4 and cleaved PARP, resulting in cell cycle arrest and increased apoptosis. This combination therapy showed a trend of superior efficacy in the imatinib‐sensitive group [81].

**FIGURE 3:**
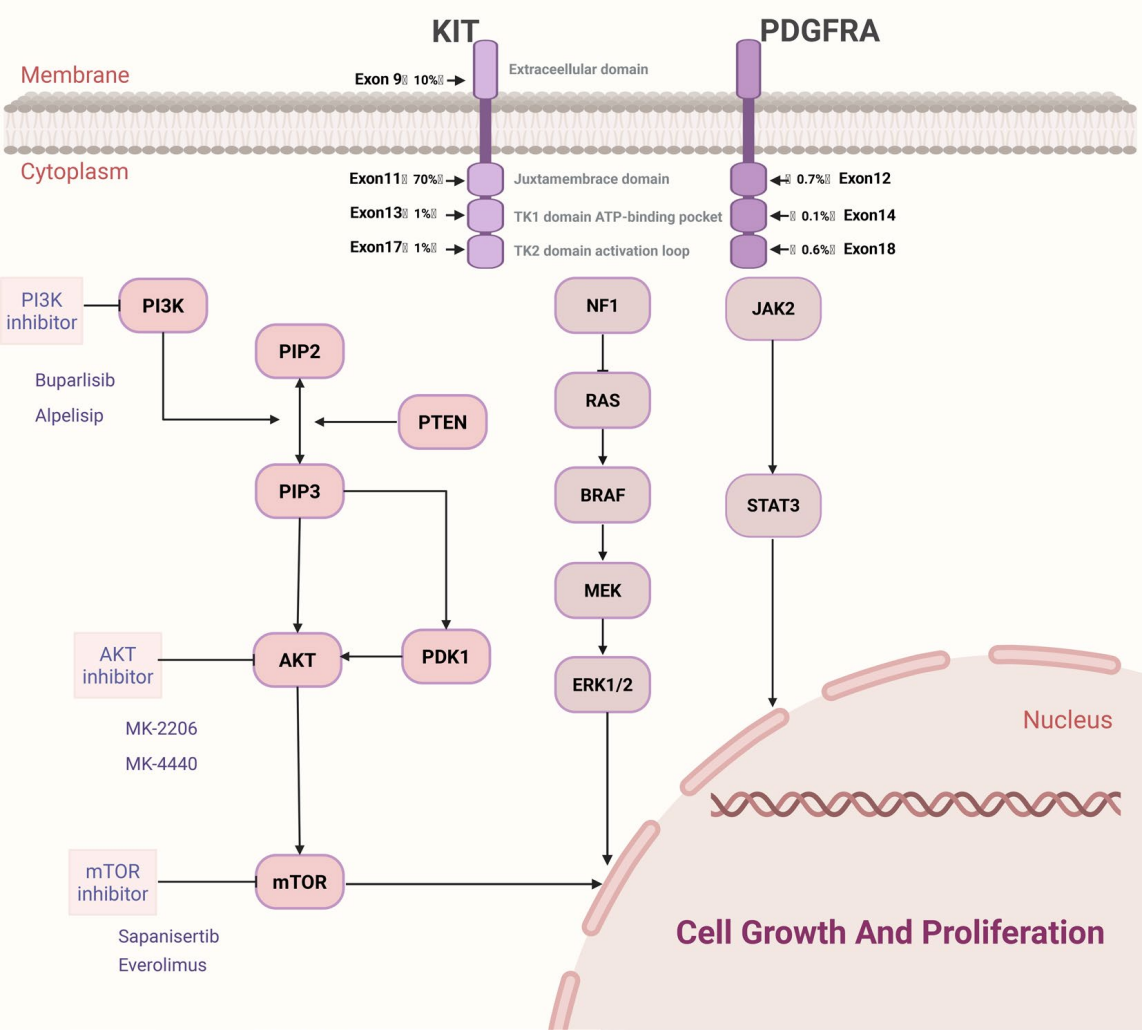
PI3K/AKT/mTOR signalling pathway in GIST. KIT mutations most frequently occur in exon 11, with additional mutations in exons 9, 13, and 17. The subsequent intracellular signalling network is centrally mediated through the PI3K/AKT/mTOR pathway, which promotes tumour growth and proliferation. Therapeutic strategies are increasingly focused on deploying specific inhibitors against key signalling nodes—such as PI3K, AKT, mTOR, and the RAS/MAPK pathway—to suppress this oncogenic cascade.

mTOR is central in regulating cell growth and proliferation, and it plays a key role in angiogenesis [82]. Activation of the mTOR pathway is characteristic of PDGFRA‐mutant and wild‐type GISTs, indicating that mTOR inhibitors may offer therapeutic potential for primary imatinib‐resistant GISTs [61]. A study involving 108 GIST patients revealed that mTOR signalling was highly activated in both PDGFRA‐mutant and wild‐type cases, further supporting the potential therapeutic value of mTOR inhibitors for primary‐resistant GISTs [83]. Sapanisertib (TAK‐228/MLN0128), a second‐generation, ATP‐competitive mTOR inhibitor (Type I) targeting both mTORC1 and mTORC2 inhibits mTORC1 and mTORC2, leading to a strong decrease in AKT and 4E‐BP1 phosphorylation. It exhibits potent anti‐proliferative effects in both imatinib‐sensitive and resistant cell lines (including KIT‐negative GIST) and shows moderate synergistic effects when combined with imatinib [84]. Pang et al. [85] demonstrated that DEPDC5 inactivates mTORC1 signalling, thereby inhibiting the phosphorylation of p70S6K and S6, which results in reduced GIST cell proliferation and subsequent cell cycle arrest. Both in vitro and in vivo studies have confirmed that the combination of everolimus (an mTOR inhibitor) and imatinib effectively inhibits tumour growth and metabolic activity [85, 86]. In phase I‐II clinical trials, this combination has shown favourable tolerability and exhibited strong synergistic effects [87] (Figure 3).

### 3.2 Role of FGF Signalling Pathway

Fibroblast growth factor receptors (FGFRs) are a family of RTKs comprising five members (FGFR1–5), with FGF2 being the most extensively studied in this context [88, 89, 90]. Activation of the FGFR signalling pathway may synergize with VEGFR‐ and PDGFR‐mediated pathways to enhance tumour angiogenesis [91].

**FIGURE 4:**
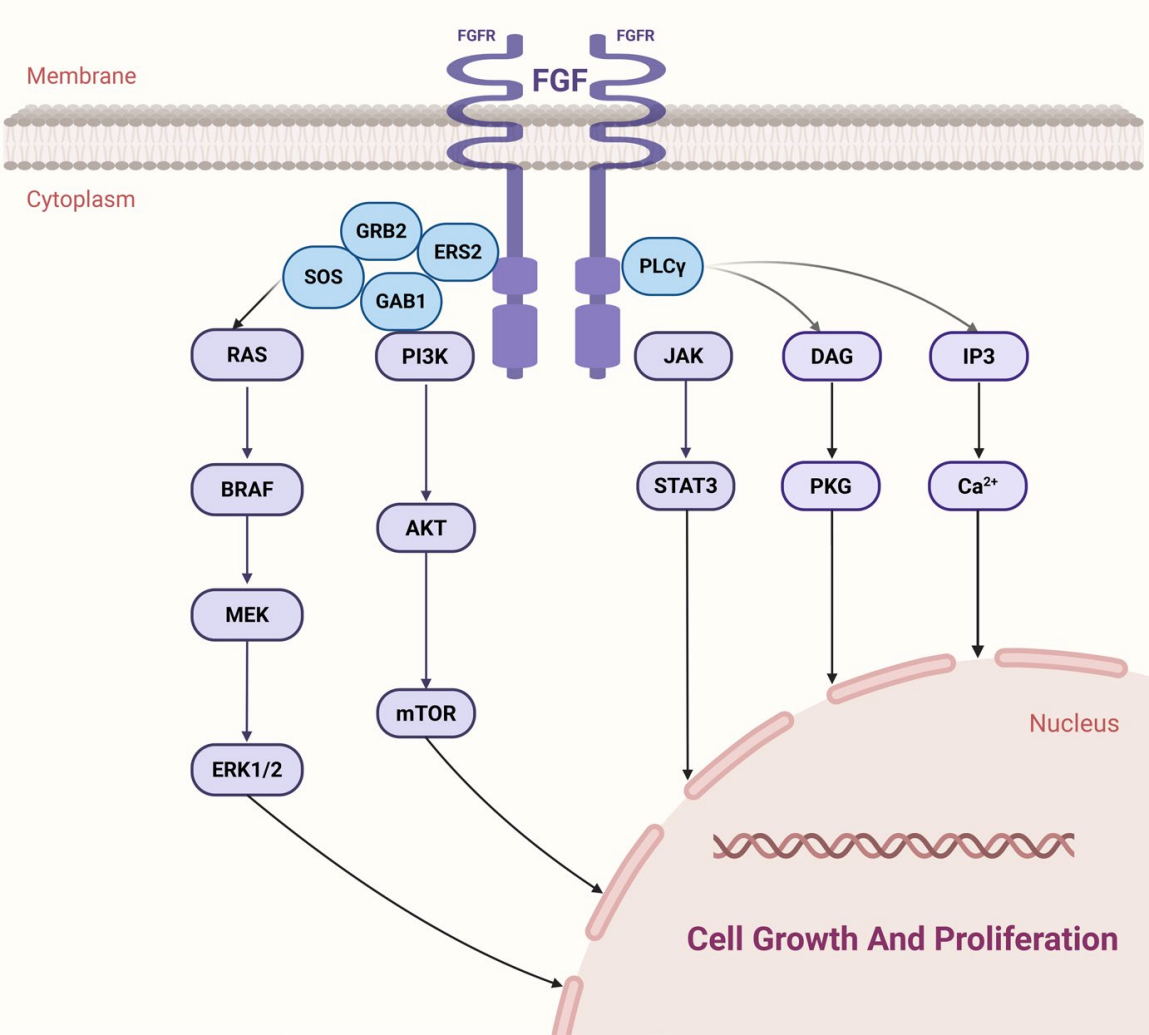
FGF signalling pathway: The FGF signalling pathway demonstrates significant cross‐talk with established oncogenic pathways in GIST, particularly through its parallel activation of the PI3K/AKT/mTOR and RAS/MAPK cascades. These pathways are similarly engaged by mutant KIT/PDGFRA receptors in GIST pathogenesis, creating potential bypass mechanisms that may contribute to tyrosine kinase inhibitor resistance. The convergence of FGF signalling with core GIST driver pathways at multiple nodes—including PI3K, AKT, mTOR, and ERK—suggests that co‐targeting FGF signalling alongside KIT/PDGFRA could represent a promising therapeutic strategy, particularly in cases where monotherapy resistance develops through alternative pathway activation.

The FGFR pathway has been well‐documented in GISTs and is closely linked to imatinib resistance. FGF2 binds to and activates its receptor FGFR, thereby mediating the reactivation of the KIT and MAPK pathways [92]. FGF2 is highly expressed in both imatinib‐resistant GIST cells and tumour tissues of patients, and its elevated expression serves as an independent prognostic factor for poor outcomes in GISTs [93, 94]. Imatinib induces autocrine activation of the FGFR signalling pathway in GISTs by overproducing and secreting the FGF‐2 ligand [91]. BGJ398, a selective FGFR1‐3 inhibitor(Type I) eliminates ERK activity rebound and, in combination with imatinib, effectively inhibits MAPK phosphorylation in both in vitro and in vivo experiments, promoting antitumor proliferation and apoptosis, thereby resensitizing imatinib‐resistant GISTs to imatinib [95, 96, 97]. KIT inhibition leads to inactivation of the ERK signalling pathway and removal of negative feedback on the FGF signalling pathway, thereby reducing dependence on KIT [96]. In a recent study, Sergei and colleagues discovered that the FGF2/FGFR pathway regulates VEGF‐A/VEGFR signalling in imatinib‐resistant GIST cells. The presence of anti‐FGF2 mAb eliminated VEGFR signalling activation in GISTs, and combining FGFR inhibitors with VEGFR inhibitors such as sunitinib or regorafenib showed strong synergy in resistant cells [98]. Notably, FGFR inhibition weakens the repair of DNA double‐strand breaks (DSBs), reduces viability in imatinib‐resistant GIST cells, promotes apoptosis, and restores sensitivity to imatinib, while having no effect on imatinib‐sensitive GIST cell lines [95, 99] (Figure 4).

Examples of non‐selective multikinase inhibitors targeting FGFR include regorafenib, dovitinib (TKI258, Type I), masitinib (AB1010, Type I/II), ponatinib (AP24534, Type II), and pazopanib (Type II/VEGFR2‐focused) [100, 101]. Nintedanib (BIBF 1120, a Type I/II multi‐kinase inhibitor targeting VEGFR, PDGFR, FGFR, and FLT3), the first FDA‐approved drug for idiopathic pulmonary fibrosis, addresses resistance caused by KIT mutations and ERK activation due to FGF upregulation [102]. A phase Ib clinical trial conducted by Memorial Sloan Kettering Cancer Center (MSKCC) combined imatinib with BGJ398, an FGFR1‐4 inhibitor, enrolling 16 patients. Of these, 25% achieved sustained disease stability for at least 32 weeks. However, the combination's toxicity significantly hindered the study's ability to meet its primary endpoint [103].

In summary, these findings strongly suggest that the FGF signalling pathway plays a critical role in both adaptive and acquired resistance to imatinib in GIST biology. Although FGF inhibitors show potential as strategies to overcome imatinib resistance, selective FGF inhibitors are still lacking.

### 3.3 Non‐Coding RNAs

Noncoding RNAs (ncRNAs) are classified into two categories based on their length: long ncRNAs (lncRNAs, longer than 200 nucleotides) and short ncRNAs (sncRNAs, shorter than 200 nucleotides, typically 20–30 nt). ncRNAs regulate cancer development by modulating key signalling pathways involved in cell proliferation, apoptosis, invasion, and metastasis [104, 105, 106]. Among the different types of ncRNAs, miRNAs, circRNAs, and lncRNAs have emerged as critical cancer regulators.

miRNAs are single‐stranded ncRNAs, approximately 18–22 nt in length, that bind partially to the 3′ untranslated region (UTR) of target mRNAs, causing mRNA degradation and/or translation repression. These molecules act as post‐transcriptional regulators of gene expression [107, 108]. miRNAs are pivotal in regulating KIT expression, with dysregulation linked to gene mutations and chromosomal alterations. They play a significant role in the initiation, progression, recurrence, metastasis, and resistance of GISTs [70, 109, 110, 111]. For example, miR‐148b‐3p can directly target KIT mRNA by binding to the 3′ UTR of KIT at nucleotides 1378–1393 and 1639–1656 [70]. Akaçakaya et al. compared the miRNA expression profiles of 17 GIST patients (10 sensitive to imatinib, 7 resistant) using microarray and RT‐PCR techniques. They identified miR‐125a‐5p as being highly expressed in resistant tissues and cells, and it promotes imatinib resistance by inhibiting the expression of PTPN18 [112]. In vitro and in vivo experiments have shown that miR‐30a downregulates beclin‐1, regulates autophagy, and enhances GIST cell sensitivity to imatinib [113]. Literature has reported differences in miRNA expression profiles between PDGFRA D842V and non‐D842V mutant GISTs. PDGFRA D842V GISTs display distinct immunological fingerprints and miRNA profiles [114], which may contribute to the primary resistance to imatinib observed in this subtype. Some miRNAs regulate oxidative phosphorylation (OXPHOS) by targeting the expression of mitochondrial electron transport chain complexes. For instance, downregulation of miRNA‐483‐3p in imatinib‐treated GIST cells inhibits the protein expression of mitochondrial respiratory complex II, and inhibition of this miRNA leads to a partial increase in OXPHOS after imatinib treatment [115].

Long noncoding RNAs (lncRNAs) have unusual 5′‐7metG caps and Poly‐A tails like mRNA. They are involved in various biological processes, regulating many cellular processes such as invasion, survival, resistance, and metastasis [116, 117, 118]. Compared to primary GIST tissues, many lncRNAs show differential expression in recurrent GIST tissues [119]. The human lncRNA HOTAIR (HOX transcript antisense intergenic RNA) is a 2364 bp RNA and serves as a typical example of a carcinogenic transcription factor lncRNA [120, 121]. HOTAIR is upregulated in recurrent GIST tissues and is associated with imatinib sensitivity, enhancing its effect by regulating autophagy [120, 122]. Knockdown of the CCDC26 lncRNA induces imatinib resistance in GIST cells through downregulation of c‐KIT expression [123, 124]. These studies suggest that lncRNAs, as noncoding RNAs, are closely associated with GIST resistance, which could serve as a novel therapeutic strategy for GIST.

ncRNAs have become important players in imatinib resistance in GISTs. However, our understanding of ncRNAs in cancer is still quite limited. Increasing research is revealing the functional roles of miRNAs in GISTs, as they are involved in tumour growth, metastasis, and treatment resistance, holding significant diagnostic, therapeutic, and prognostic value for GIST patients. However, further high‐quality clinical and basic research is needed to translate the current findings into clinical practice.

### 3.4 Autophagy

Autophagy is the process in which cytoplasmic material is enclosed in double‐membraned vesicles, forming autophagosomes that fuse with lysosomes for digestion. It is a self‐degradation and recycling process of intracellular components, which is crucial for maintaining eukaryotic cell homeostasis and overall cell health. Autophagy is responsible for removing cellular stressors and harmful substances, ranging from misfolded or aggregated proteins and lipid droplets to entire damaged or aged organelles. When necessary, autophagy utilises the byproducts as energy substrates or precursors for structural rebuilding [125]. In mammalian cells, autophagy is typically classified into three types based on the pathway through which substrates enter the lysosome: macroautophagy, microautophagy, and chaperone‐mediated autophagy (CMA) [126, 127, 128]. Autophagy plays a complex, environment‐dependent role in cancer, acting as a double‐edged sword. It can suppress tumour initiation and reduce genomic instability but may also promote the survival of established tumours [129]. Autophagy influences tumorigenesis and progression by supporting nutrient recycling and metabolic balance [130]. Constitutive autophagy in tumour chemotherapy can prevent chemotherapy‐induced cell death, leading to resistance and tumour recurrence [131].

In 2008, Miselli and collaborators reported the first strong indirect evidence of autophagy in GISTs. This study analysed the morphological, biochemical, and immunophenotypic characteristics of 13 surgically resected GIST patient samples, 11 of which were treated with imatinib. They found that the imatinib response in GIST patients is maintained through autophagy, rather than apoptosis, based on the expression of autophagy‐related proteins such as beclin1, PI3KI, and PI3KIII in the surgical samples [132]. KIT protein is partially degraded by the autophagy‐lysosome system, influencing its phosphorylation and total protein levels [27]. Following imatinib treatment in GIST cells, phosphorylation levels of mTOR and its primary target S6K are reduced, activating the autophagy signalling pathway and upregulating BECN1 levels [27]. In imatinib‐resistant cell lines, with or without chloroquine (CQ) treatment, autophagy markers ATG5 and LC3‐II/I are elevated, and imatinib induces protective autophagy in GISTs [129]. Imatinib‐induced autophagy activation is also a critical survival pathway for dormant GIST cells [25, 133]. Neurotrophic GDNF protects GFRA1‐positive tumour cells from apoptosis under oncogenic and metabolic stress through the autophagy‐lysosome system and the MCOLN1/Ca2+/TFEB pathway, promoting GIST cell dormancy [27]. Inhibition of autophagy using lysosome‐targeting drugs can reduce imatinib resistance in GISTs both in vitro and in vivo. Chloroquine (CQ) and its derivative hydroxychloroquine (HCQ), which inhibit autophagy by alkalinizing the lysosome and preventing autophagosome degradation, synergize with imatinib, enhancing GIST cell death and delaying the growth of surviving GIST cells [134, 135]. Additionally, lncRNAs can regulate autophagy in cancer cells, and they have been extensively studied in GISTs, emerging as a promising strategy to overcome resistance [113, 120, 122, 130, 131, 136].

**FIGURE 5:**
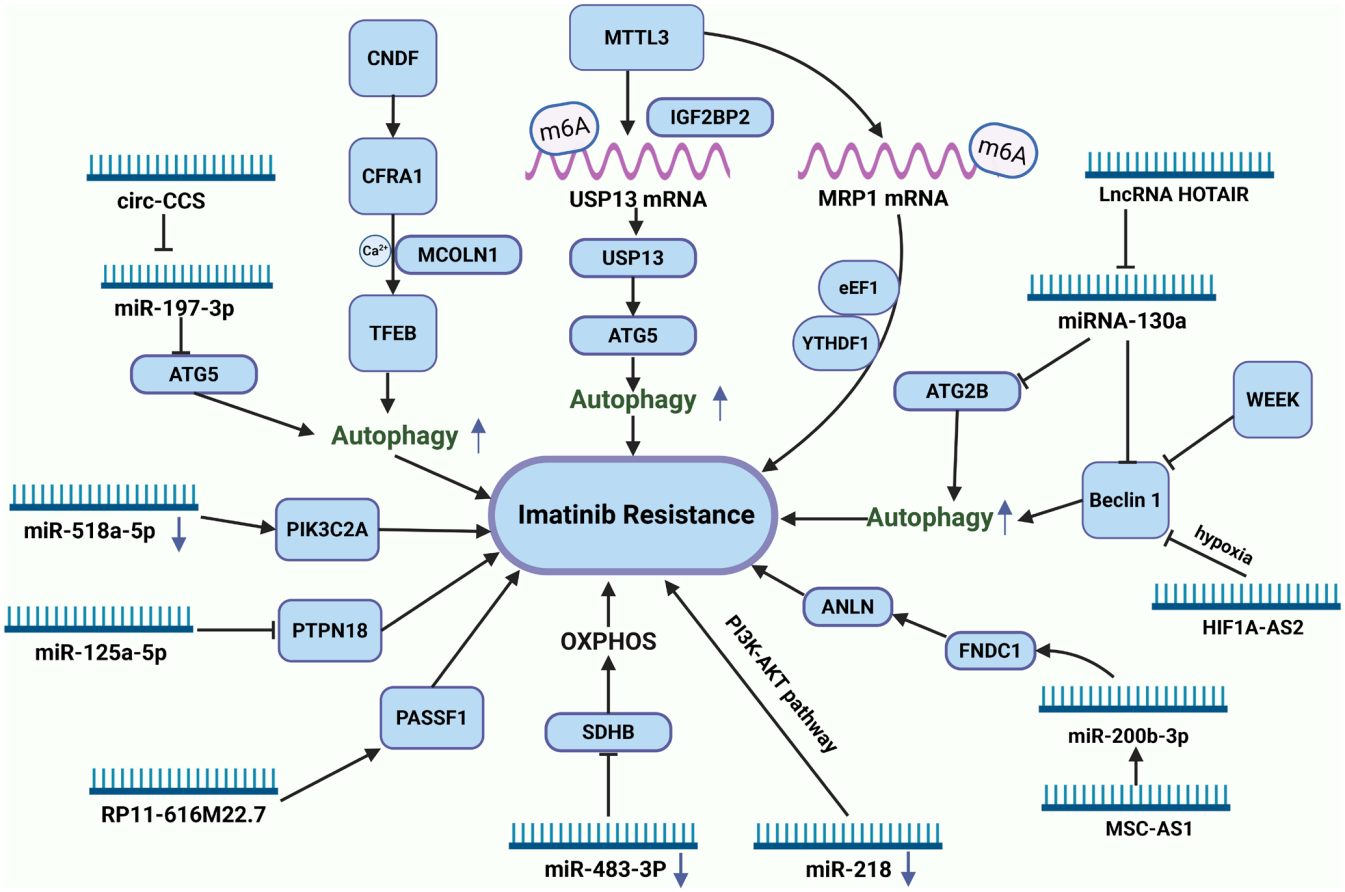
The mechanism of ncRNA‐mediated imatinib resistance. ncRNAs are abnormally expressed in GIST and participate in imatinib resistance by regulating different target genes and biological processes. Different ncRNAs can act as upstream signals to target key molecules and accelerate the autophagy process.

Based on these studies, it can be concluded that autophagy plays a protective role in imatinib‐induced GIST cell death. Modulating autophagy affects tumour cell survival, response to treatment, and overall therapeutic outcomes, making it a potential therapeutic target for overcoming resistance. Looking forward, with the continued advancement of basic autophagy research, more autophagy modulators will be discovered and applied in disease treatment (Figure 5).

## 4 The Role of Immunotherapy in Imatinib Resistance

Activating and boosting the immune system to eliminate tumour cells has emerged as a widely used antitumour approach in recent years. The GIST microenvironment is characterised by extensive infiltration of tumour immune cells, with macrophages and T lymphocytes being the most common. Additionally, there are small amounts of B cells, natural killer (NK) cells, dendritic cells (DC), γδT cells, neutrophils, eosinophils, and mast cells, all of which play vital roles in tumour monitoring [137, 138]. GISTs exhibit high levels of CD8+ T cell infiltration, which correlates with favourable clinical outcomes, including recurrence‐free survival (RFS) and overall survival (OS). This infiltration also contributes to better responses to imatinib treatment [139, 140]. PDGFRA‐mutant GISTs, compared to KIT‐mutant GISTs, have more T cells and exhibit a stronger tumour‐killing ability, while wtGISTs show less immune infiltration [52, 141, 142].

Immune checkpoint inhibitors, such as PD‐1/PD‐L1 and CTLA‐4, are key immune suppressive targets in tumour escape mechanisms. Compared to untreated tumours, CD4+ T cells in sensitive tumours express higher levels of PD‐1, while CD8+ T cells in imatinib‐resistant tumours also exhibit elevated PD‐1 expression [143]. PD‐L1 expression can inhibit immune cell attack, particularly by suppressing CD8+ T cells. Blocking the PD‐1/PD‐L1 interaction enhances the antitumour effects of imatinib by rescuing exhausted CD8+ T cells in PD‐L1‐positive GIST cells, particularly through the PI3K/Akt/mTOR pathway [71]. Following imatinib treatment, the GIST microenvironment changes, primarily due to the activation of CD8+ T cells and dendritic cells, leading to an initial boost in the immune response. However, with prolonged imatinib treatment, the abundance of dendritic cells and CD8+ T cells within the tumour decreases, which suppresses the immune response against tumour cells [42]. In the GIST KitV558Δ/+ mouse model, neither anti‐PD1 nor anti‐PD‐L1 antibodies alone had an impact on GIST. However, combining anti‐PD‐1 antibodies with imatinib significantly reduced tumour growth by boosting the effector function of CD8+ T cells [144].

Current research is focused on investigating the synergy between immune checkpoint inhibitors (ICIs) and conventional targeted therapies in treating GIST patients. In phase I/II clinical trials for patients with advanced resistant GIST, the combination of PD‐1 antibodies and imatinib demonstrated good safety. However, PFS and overall survival (OS) were 2.3 and 9.5 months, respectively, both significantly lower than the PFS achieved with sunitinib and regorafenib [145]. In a randomised phase II study, the safety and tolerance of nivolumab (N) alone and in combination with ipilimumab (N + I) were assessed. The results indicated good safety, with PFS of 11.7 weeks for N and 8.3 weeks for N + I, both lower than the PFS reported for sunitinib and regorafenib in phase III trials (8.5 and 4.8 months). However, it is important to note that two wild‐type GIST patients in this trial achieved partial remission (PR) [146]. Another phase Ib clinical trial assessed the efficacy of imatinib combined with ipilimumab in advanced GIST patients. The study found that only one wild‐type GIST patient benefited from this combination, showing partial remission (PR) [143]. Immunotherapy seems to be effective for KP‐wtGISTs, but more research is required to explore its potential [142, 147].

Although immune‐based therapies have seen strong development as key treatments for various cancers in recent years, stricter clinical trials are necessary to establish their clinical efficacy in GISTs. Immune checkpoint inhibition has radically changed the treatment of several malignancies. Despite the strong biological rationale, immunotherapy has yet to provide significant benefits for advanced GIST patients. However, it would be premature to conclude that immunotherapy is ineffective, as a small number of advanced GIST patients have achieved stable disease (SD) or partial remission (PR) with ICIs or combination therapy. Undoubtedly, the exploration of immunotherapy for GIST remains in its early stages, with ongoing controversies and suboptimal results. Further research is urgently needed to refine this approach (Table 1).

**TABLE 1:** GIST immunotherapy clinical trials registered on https://clinicaltrials.gov/.

Drugs	Therapy targets	Trail phase	Status	Clinical trials gov ID	Study completion year
Peginterferon α‐2b + IM	Immunomodulator/TKI	II	Terminated	NCT00585221	2009
Dasatinib + Ipilimumab	TKI/CTLA‐4	I	Completed	NCT01643278	2016
Pazopanib+IM	TKI	II	Completed	NCT01323400	2016
PLX3397 + PembrolizumaB	PD1/CSF1R	1/2a	Terminated	NCT02452424	2018
Epacadostat + Pembrolizumab	PD1	II	Terminated	NCT03291054	2020
PDR001 + IM	PD1/TKI	I/II	Completed	NCT03609424	2021
PDR001 + IM	PD1/TKI	Ib/II	Completed	NCT03609424	2021
Nivolumab + Ipilimumab	PD1/CTLA‐4	II	Completed	NCT02880020	2022
Ipilimumab + IM	CTLA‐4/TKI	I	Completed	NCT01738139	2023
Avelumab + Axitinib	PD‐L1	II	Recruiting	NCT04258956	2023
Nivolumab + Ipilimumab	PD1/CTLA‐4	II	Completed	NCT02500797	2023
TNO155 + partalizumab OR TNO155 + Ribociclib	SHP2/PD1	Ib	Terminated	NCT04000529	2024
Atezolizumab + IM	PD‐L1/TKI	II	Recruiting	NCT05152472	Estimated 2027
Nivolumab +Ipilimumab	PD1/CTLA‐4	II	Active	NCT02834013	Estimated 2026

Abbreviation: IM, imatinib.

## 5 Tumour Metabolism and Imatinib Resistance

Cancer cells reprogram their metabolism to support growth, metastasis, and survival. They exhibit increased dependence on glycolysis, with enhanced glucose uptake and lactate fermentation to satisfy the high anabolic needs for cancer cell proliferation [148]. Metabolic reprogramming refers to the changes and adjustments in metabolic pathways that tumour cells undergo during development, driven mainly by the tumour tissue's unique chemical microenvironment. Similarly, during imatinib therapy, cancer cell metabolism is reprogrammed to adapt to the altered environment. Thus, understanding cancer phenotypes through metabolomics may provide a promising approach to targeting these metabolic alterations and overcoming resistance.

Cancer cells often exhibit a dependence on glycolysis, referred to as the ‘Warburg effect’. Abnormal glycolysis plays a crucial role in cancer resistance [149]. Reprogramming of glucose metabolism in cancer cells triggers DNA repair and immune suppression within the tumour microenvironment, contributing to resistance. GISTs show high metabolic activity and glucose uptake, as observed in 18F‐FDG‐PET scans. A decrease in glucose uptake is observed within 24 h of imatinib treatment, coinciding with the emergence of a quiescent cell phenotype, which could represent another mechanism of imatinib resistance in GISTs [150]. After imatinib treatment, oxidative phosphorylation (OXPHOS) in mitochondrial respiratory complexes II, III, and V is upregulated. VLX600, a mitochondrial OXPHOS inhibitor, suppresses OXPHOS, increases GLUT1 expression, and enhances AKT signalling in vitro [150, 151]. GIST cells resistant to imatinib exhibit elevated oxidative phosphorylation and higher glycolytic rates [151, 152]. SQSTM1 plays a role in glucose metabolism reprogramming, with elevated SQSTM1 expression in GISTs inhibiting the response to imatinib mesylate. Higher SQSTM1 expression is negatively associated with prognosis [153]. Glucose transporter (GLUT) is a critical rate‐limiting factor in glucose metabolism, playing a key role in cancer cell metabolism and being associated with poor prognosis [154, 155]. Following low‐dose imatinib treatment, GLUT‐1 levels in GIST cells decrease in a dose‐dependent manner, while GLUT‐1 levels in imatinib‐resistant cells increase proportionally with the dose. When the GLUT‐1 inhibitor WZB117 is used alone or in combination with imatinib to treat GIST‐sensitive and resistant cells, the combined therapy significantly suppresses cell proliferation [156]. These findings suggest that the combination of the GLUT‐1 inhibitor WZB117 and imatinib may reverse imatinib resistance in resistant cells. Therefore, glycolysis inhibitors could increase cancer cell sensitivity to chemotherapy and radiotherapy, helping to overcome treatment resistance.

However, the clinical application of glycolysis inhibition to counteract resistance remains limited. Future studies should focus on identifying key metabolic changes that contribute to resistance and exploring their potential as drug targets to improve GIST sensitivity to targeted therapies and combat resistance.

## 6 Conclusion and Future Directions

Targeted therapies can accurately identify specific biomolecules involved in the onset and progression of diseases, particularly cancer, and inhibit their function or induce cell death. The discovery of TKIs in the past two decades has drastically transformed the treatment of GISTs. The FDA has approved seven drugs for the treatment of advanced GISTs: imatinib, sunitinib, regorafenib, ripretinib, avapritinib, larotrectinib, and entrectinib. These inhibitors vary in their specificity and binding mode (Type I vs. Type II), which underlies their distinct efficacy and resistance profiles against different GIST mutations. However, TKI therapies are insufficient for long‐term disease control, and the development of resistance introduces new challenges in GIST treatment. Although these therapies initially provide clinical benefit, resistance inevitably emerges, undermining their long‐term efficacy. The precise mechanisms underlying resistance remain largely unclear. Understanding resistance mechanisms is essential to enhancing the clinical efficacy of imatinib. Exploring combination strategies that involve targeted therapies and other treatment modalities could potentially improve outcomes. Additionally, new therapies need to be developed. However, current evidence suggests that translating in vitro anticancer activity into clinical efficacy remains a significant challenge, as many promising in vitro observations fail to result in clinical benefits.

## Author Contributions

**Nuerbiye Abudurexiti:** visualization (equal), writing – original draft (equal). **Yidan Lou:** conceptualization (equal). **Mengqi Wu:** conceptualization (equal). **Yingchen Huang:** writing – review and editing (equal). **Shan Huang:** conceptualization (equal). **Kaibo Guo:** visualization (equal). **Song Zheng:** supervision (equal), writing – review and editing (equal).

## Conflicts of Interest

The authors declare no conflicts of interest.

## Data Availability

Data derived from public domain resources.
